# Comparative Analysis of Hypertension Guidelines: Unveiling Consensus and Discrepancies in Lifestyle Modifications for Blood Pressure Control

**DOI:** 10.1155/2023/5586403

**Published:** 2023-12-12

**Authors:** Yi Wang, Yanxiang Liu, Lu Liu, Liqiong Hong, Huimin Chen

**Affiliations:** ^1^Second Affiliated Hospital, School of Medicine, Zhejiang University, Hangzhou 310000, China; ^2^Fuwai Hospital, Chinese Academy of Medical Sciences and Peking Union Medical College, Beijing 100000, China

## Abstract

**Background:**

Hypertension is a major global health concern, and lifestyle modifications have been recommended as first-line treatment for hypertension in many guidelines. However, different guidelines may recommend different types of lifestyle adjustment, and it is unclear which ones are most effective. In this review, we compared hypertension guidelines to identify any differences and/or consensus in content, efficacy, and timing of initiation of lifestyle modifications.

**Methods:**

We conducted a search of databases to identify hypertension guidelines available in English. We extracted and compared information about lifestyle modifications recommended by the guidelines.

**Results:**

Five hypertension guidelines from America, Europe, the UK, Canada, and the International Society of Hypertension are included. They all recommend diet adaptation, sodium reduction, alcohol restriction, physical exercise, and weight reduction. Other lifestyle interventions emphasized by some guidelines, such as potassium supplementation, smoking cessation, and stress management, are not recommended by all the five guidelines. Among lifestyle changes, the dietary approaches to stop hypertension (DASH) diet may be considered the most effective treatment for reducing blood pressure. These guidelines recommend that for patients with high-normal blood pressure or grade 1 hypertension without high risk factors, lifestyle medicine should be used first for 3–6 months, if blood pressure is still not controlled, then start medication. For those patients who need drug treatment, lifestyle changes can also enhance the effects of antihypertensive therapy.

**Conclusion:**

Lifestyle modifications are crucial in the treatment of hypertension and should be recommended to most hypertensive patients. Among these lifestyle interventions, diet adaptation containing low sodium and alcohol restriction may be the most effective in reducing blood pressure. Physical exercise and weight reduction are also recommended. In some cases, lifestyle modifications should be tried first. They may also enhance the effects of antihypertensive drugs in other patients.

## 1. Introduction

Hypertension is recognised as the leading contributor to the global burden of disease, causing approximately 9.4 million deaths per year [1]. It is a major risk factor for mortality and disability and is associated with an increased risk of cardiovascular disease (CVD) events such as coronary heart disease, heart failure, and stroke [2]. Numerous studies have shown that lower blood pressure (BP) is associated with fewer CVD events and reduced mortality [3, 4].

Lifestyle modifications that adjust diet, physical activity, weight, alcohol consumption, and other aspects have been recommended as the first-line treatment for hypertension in guidelines. These lifestyle changes have been shown to lower blood pressure [5–9], and their hypotensive effects are partially additive and may enhance the efficacy of medication [2].

However, due to differences in economic level, social culture, and medical resources of various countries and regions, different guidelines may recommend different types of lifestyle modifications. This may cause confusion among clinical staff: Which methods are recommended by most guidelines? Are there differences in the efficacy of different lifestyle changes? How can we balance lifestyle therapy and drug therapy?

The purpose of this review is to compare lifestyle modifications recommended by hypertension guidelines from different countries and provide information on any differences and/or consensus in content, efficacy, and timing of initiation of lifestyle modifications for hypertension.

## 2. Materials and Methods

### 2.1. Literature Search

Researchers searched the PubMed and EMBASE databases. To ensure the inclusion of the latest research findings, only guidelines published within the last five years (from January 1, 2017, to December 1, 2022) were selected. The search keywords used were “hypertension” or “high blood pressure” and “guidelines”. The following search strategy was used for PubMed:

  #1 Guideline [Publication Type]
  #2 Hypertension [Title] OR Blood Pressure, High [Title] OR Blood Pressures, High [Title] OR High Blood Pressure [Title] OR High Blood Pressures [Title]
  #3 #1 AND #2
  #4 2017/01/01:2022/12/01 [Date - Publication]
  #5 #3 and #4

### 2.2. Inclusion and Exclusion Criteria

The inclusion criteria for this review were guidelines written in English and published from 2017 onward. Guidelines focussing on special populations such as children, adolescents or pregnant women, and those not related to arterial blood pressure, such as pulmonary hypertension, were excluded. Additionally, consensus statements, position papers, older versions of guidelines, and those not containing recommendations for lifestyle medicine were also excluded.

Two reviewers independently screened the titles and abstracts of all potentially eligible guidelines and performed a full-text review to identify guidelines that met all inclusion criteria.

## 3. Results

**Figure 1:**
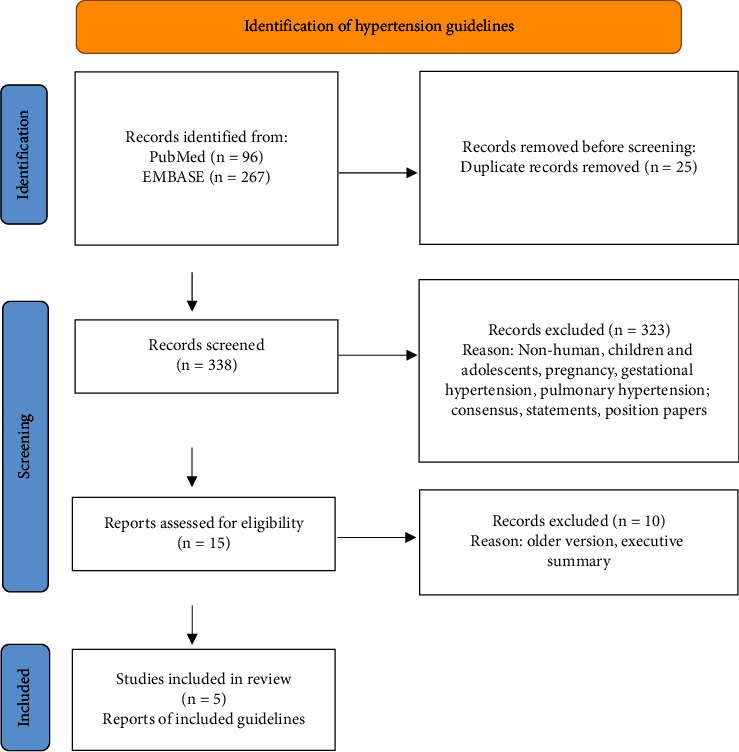
Retrieval process.

We included adult hypertension guidelines from 5 countries or associations: America, Europe, UK, Canada, and the International Society of Hypertension, abbreviated 2017 ACC [10], 2018 ESC [11], 2019 NICE [12], 2020 CAN [13], and 2020 ISH [14], respectively. For Canada, we selected the more comprehensive guideline on hypertension management [13], as lifestyle management in the guideline on resistant hypertension [15] was referenced from another guideline. The retrieval process is shown in Figure 1.

Lifestyle medicine is a crucial approach to prevent and manage hypertension, either alone or in combination with medication. Every guideline mentions diet adaptation with low sodium, physical exercise, weight reduction, and alcohol restriction. Three guidelines recommend potassium supplementation [10, 13, 14] and smoking cessation [11, 12, 14], while two guidelines recommend stress management [13, 14]. Other contents of the guidelines are presented in Table 1. Various other lifestyle interventions have been reported to lower blood pressure, but the supporting clinical trials' extent or quality is less persuasive or adequate. Therefore, we do not take them as recommended advice. The recommendations of the guidelines are summarized in Table 1.

### 3.1. Diet Adaptation

The United States, Canada, and ISH clearly suggest adopting a DASH diet characterised by high amounts of fruits and vegetables, low-fat dairy foods, whole grains, nuts, fish, and poultry but reduced amounts of red meat, beverages, saturated fat, sweets, and snacks. The DASH diet is the most effective diet proven for lowering blood pressure among lifestyle approaches for treating hypertension [10] or prehypertension [16]. It was associated with an overall reduction of approximately 11 mmHg in hypertensive and 3 mmHg in nonhypertensive adults [5]. The effect size increased significantly when combined with weight loss [17] or a reduction in sodium intake [18]. Other diets, particularly the Mediterranean diet, have been shown to reduce BP and are recommended by European guidelines [11]. The Mediterranean diet is similar to the DASH diet pattern, and it is advised to eat a healthy balanced diet containing vegetables, legumes, fresh fruits, low-fat dairy products, whole grains, fish, and unsaturated fatty acids (especially olive oil) and to limit consumption of red meat and saturated fatty acids [19]. However, the Mediterranean diet includes moderate consumption of alcohol, mostly wine with meals [11]. The recommendations of the guidelines are presented in Table 2.

### 3.2. Sodium Reduction

There is strong evidence for the association between high salt intake and increased blood pressure [20]. Globally, the usual sodium intake is between 3.5–5.5 g per day (which corresponds to 9–12 g of salt per day), with marked differences between countries and even between regions [11]. An early meta-analysis showed that a modest reduction in sodium intake of 1 g/d led to a reduction in systolic blood pressure (SBP) by 3.1 mmHg in hypertensive individuals and by 1.6 mmHg in normotensive individuals [21]. Europe and Canada recommend limiting salt intake to no more than 5 g per day (equivalent to approximately 2000 mg of sodium). The United States recommends limiting salt intake to no more than 3.75 g per day (equivalent to approximately 1500 mg/d of sodium), and it can be stricter, which can reduce blood pressure by 5/6 mmHg in hypertensive individuals or 2/3 mmHg in nonhypertensive individuals [10]. This effect can be more than double in more susceptible individuals, those with hypertension, and those concurrently on the DASH diet [22] or following a weight loss intervention. NICE and ISH did not give specific numbers for salt intake. It is difficult in the real world to vigorously reduce sodium intake to <2 g/d. It is estimated that 80% of daily salt intake comes from processed foods; therefore, the guideline recommends more basic food consumption for optimal sodium restriction [23]. The recommendations of the guidelines are presented in Table 3.

### 3.3. Alcohol Restriction

Heavy drinking induces high blood pressure and can also cause stroke, alcoholic cardiomyopathy, atrial fibrillation, and nocturnal sleep apnea, as well as cancer, increasing mortality [9, 24]. However, some epidemiological studies have suggested a J-shaped or U-shaped relationship between alcohol consumption and cardiovascular disease risk, indicating that moderate drinkers have a lower risk of myocardial infarction than nondrinkers [25, 26]. Guidelines uniformly recommend reducing or limiting alcohol consumption, although there are some variations in the recommended amount. In the United States, men are advised to consume no more than 28 g of pure alcohol per day (equivalent to approximately 148 ml of wine or 355 ml of beer), while women should limit themselves to 14 g per day. The ISH recommends 20 g per day for men and 15 g per day for women. In Europe, the recommendation is to drink no more than 1750 ml of wine or 875 ml of beer per week for men and women. In Canada, the recommendation is to consume no more than two drinks per day. The specific recommendations of the guidelines are presented in Table 4.

### 3.4. Weight Reduction

There is definite evidence for a relationship between obesity and increased incidence of hypertension [27, 28]. Losing weight is a core recommendation and should be achieved through a multidisciplinary approach that includes dietary adaptation and increased physical activity [8]. A review has demonstrated that the mean reductions in SBP and DBP associated with an average weight loss of 5.1 kg were 4.4 and 3.6 mmHg, respectively [29]. Although the guidelines do not form a consensus on ideal body weight, it is clear that body weight control is indicated to avoid obesity. Furthermore, BMI varies in different countries and regions. Europe and Canada recommend maintaining a healthy body weight (body weight index 18.5–25 kg/m^2^) and waist circumference (<102 cm for men and <88 cm for women). Waist circumference can be stricter in America, men <94 cm in men and women <80 cm, to reduce BP and CV risk. In patients with intense obesity, surgery for obesity (such as Roux-en-Y) can achieve improvement in blood pressure control [30]. The recommendations of the guidelines are presented in Table 5.

### 3.5. Physical Exercise

Patients with hypertension who engage in physical activity at any level have been shown to reduce cardiovascular mortality by 16%–67% [31]. Among the five guidelines, the most recommended exercise for hypertension is at least 30 minutes of moderate-intensity aerobic exercise, such as walking, jogging, cycling, or swimming, for 3–7 days a week or 90–150 minutes a week. The average reductions in SBP with aerobic exercise are approximately 2–4 mmHg and 5–8 mmHg in adult patients with normotension and hypertension, respectively [10, 32]. High-intensity aerobic exercise is controversial, as Canadian guidelines do not consider it more effective, but the ISH recommends high-intensity interval training (HIIT). Additionally, three guidelines recommend resistance/strength exercises, 2-3 times a week or 90–150 minutes a week. The ACC also mentions that isometric resistance training results in a substantial lowering of BP. In a meta-analysis, endurance, dynamic resistance, and isometric resistance exercises for at least 4 weeks significantly reduced SBP/DBP by 3.5/2.5, 1.8/3.2, and 10.9/6.2 mmHg, respectively [32]. This means that the effect of isometric resistance exercise is greater than that previously reported in dynamic aerobic or resistance training. This conclusion is backed up by other studies, which found that isometric training may lower systolic and diastolic blood pressure by 5 mmHg and 4 mmHg, respectively [33, 34]. However, which exercise is the most effective warrants further research. The recommendations of the guidelines are presented in Table 6.

### 3.6. Potassium Supplementation

Potassium interventions have been shown to effectively lower BP in some studies [35, 36], particularly in adult patients with excessive sodium intake [35]. The typical BP-lowering effect of a 60 mmol (1380 mg) administration of potassium chloride has been approximately 2 mmHg and 4-5 mmHg in adults with normotension and hypertension, respectively, although the response is up to twice as much in individuals consuming a high-sodium diet [10]. However, there are conflicting opinions among different guidelines regarding the recommendation to supplement with potassium. The United Kingdom suggests not offering calcium, magnesium, or potassium supplements as a method for reducing blood pressure, while the United States and Canada believe that hypertensive patients without hyperkalemia should properly consume potassium ions in their diet. The United States provides specific recommendations, which is 3500–5000 mg/d. For patients with chronic kidney disease (CKD), potassium intake needs to be restricted (≤2000 mg/d at stage 3b and ≤1500 mg/d at stage 4 or higher) [37]. The guidelines' recommendations are presented in Table 7.

### 3.7. Smoking Cessation

Studies using ambulatory blood pressure monitoring (ABPM) have shown that both normotensive individuals and untreated hypertensive smokers present higher daily blood pressure values than nonsmokers [38]. Smoking is second only to blood pressure in terms of risk for the global burden of disease, and quitting smoking is an effective lifestyle measure to prevent cardiovascular disease, including stroke, myocardial infarction, and peripheral artery disease [39]. Although smoking is an established risk factor for cardiovascular disease, the relationship between smoking and blood pressure remains unclear. Some studies suggest that smoking has little effect on blood pressure [40], and they have even found that blood pressure is lower in smokers [41]. However, three out of five guidelines recommend quitting smoking to reduce blood pressure. Further research is needed to determine whether quitting smoking can effectively reduce blood pressure. The guidelines' recommendations are presented in Table 8.

### 3.8. Stress Management

Patients with hypertension experience twofold or more stress compared with normotensive individuals [42]. Mental and social stress can increase the incidence of hypertension by two or more times [43]. Chronic stress has been associated with high blood pressure later in life, according to Canada and ISH guidelines, and stress management techniques such as meditation and individualized cognitive-behavioural interventions may be effective. However, some guidelines do not make recommendations or even remove advice due to insufficient evidence and the need for further research [44]. The guidelines' recommendations are presented in Table 9.

### 3.9. Other Lifestyle Medicine

Caffeine has been shown to have an acute pressor effect [31]. The NICE and ISH guidelines recommend discouraging excessive consumption of coffee and other caffeine-rich products. However, a recent systematic review of prospective cohort studies has highlighted that coffee consumption may be associated with cardiovascular benefits [31]. Additionally, consumption of green or black tea may also have a small but significant BP-lowering effect [45]. The ISH guideline also recommends moderate consumption of green and black tea, as well as other beverages such as karkadé (hibiscus) tea, pomegranate juice, beetroot juice, and cocoa. While the Canadian guideline recommends against supplementation of calcium and magnesium for the prevention or treatment of hypertension (Grade B), the ISH recommends increasing the intake of other beneficial foods and nutrients such as those high in magnesium, calcium, and potassium, including avocados, nuts, seeds, legumes, and tofu. The ISH also mentioned reducing exposure to air pollution and cold temperature. Studies have shown that the cardiovascular mortality rate during winter is higher when protective measures against the cold are inadequate [46].

## 4. Discussion

### 4.1. When Should Lifestyle Modifications Be Used for Reducing Blood Pressure?

According to research, a healthy lifestyle can prevent or delay the occurrence of high blood pressure [23]. In fact, lifestyle adjustments are considered the first-line treatment for hypertension.

For lower-risk patients with grade 1 hypertension or high-normal blood pressure, blood pressure-lowering mediation should be initiated after 3–6 months if lifestyle interventions alone are not sufficient to control blood pressure [10, 11]. The ISH suggests that if drugs are limited in availability, drug treatment could start in those aged 50–80 years when facing the above situation [14].

Lifestyle modifications can also enhance the effectiveness of antihypertensive medication. In patients with grade 2 or 3 hypertension, it is recommended that blood pressure-lowering drug treatment should be initiated alongside lifestyle interventions. For patients with grade 1 hypertension at high risk or with hypertension-mediated organ damage (HMOD), drug treatment should also be initiated simultaneously with lifestyle interventions [10, 11, 13, 14].

### 4.2. Which Is the Most Effective Lifestyle Approach in Treating Hypertension?

The DASH diet has been proven to be the most effective among lifestyle approaches for lowering blood pressure in those with hypertension [11] or high-normal blood pressure [17]. Those who concurrently follow the DASH diet can experience a reduction in blood pressure more than double that of those who do not [22]. Among the various lifestyle interventions recommended in the guidelines, the most commonly mentioned are related to diet, including dietary pattern management, reducing salt intake, and restricting alcohol consumption. Some measures with insufficient evidence, such as potassium supplementation, discouraging excessive consumption of coffee and other caffeine-rich products, and moderate intake of green tea, are all related to dietary intake. Thus, it is safe to say that diet may be the most effective lifestyle approach. The effect size can be significantly increased when combined with weight loss [18].

While, in the past, moderate-intensity aerobic exercise was believed to have the best antihypertensive effect and was the most recommended exercise in guidelines, more and more evidence suggests that isometric exercise may have a better systolic blood pressure-lowering effect. Which exercise has the most obvious effect on lowering blood pressure is worth further investigation.

### 4.3. What Is the Consensus in the Content of Lifestyle Medicine?

The five guidelines on hypertension management all recommend diet adaptation with low sodium and moderate alcohol or abstinence, physical exercise, and weight reduction.

DASH is the most commonly recommended diet, which emphasizes a high intake of fruits, vegetables, and low-fat dairy products, and provides a means to enhance the intake of potassium, calcium, magnesium, and fibre. Salt intake should be reduced to within 5 g/d, or even stricter. Alcohol intake should not exceed 20–28 g for men and 14-15 g for women per day, and binge drinking should be avoided.

In terms of physical activity, the guidelines recommend aerobic exercise, dynamic resistance, and isometric contraction training, with moderate-intensity aerobic exercise being the most recommended, lasting at least 30 minutes/day, at least 3–7 days a week.

Weight loss should be achieved by maintaining an appropriate range of body weight index (BMI), about 20–25 kg/m^2^, and waist circumference, which should be less than 102 cm for men and less than 88 cm for women.

### 4.4. What Are the Differences in the Lifestyle Medicine?

Within the spectrum of lifestyle medicine, nuances emerge as guidelines touch on weightier matters such as weight loss, the DASH diet, and physical activity. However, the quieter notes of potassium supplementation, smoking cessation, and stress management remain unevenly addressed. While we cannot conclusively dismiss the efficacy of these aspects, the literature reveals a dearth of evidence for unequivocal recommendations. As we navigate this landscape, the call for further research echoes, beckoning a deeper comprehension of the potential benefits these dimensions may unfold in the realm of hypertension management.

### 4.5. What Is the Status of New Technology in Lifestyle Medicine?

New technology has the potential to revolutionize the field of lifestyle medicine, particularly in the areas of monitoring and management of lifestyle-related conditions such as hypertension. Wearable devices, mobile applications, and intelligent platforms can provide real-time monitoring and feedback to patients, as well as personalized guidance and support for lifestyle interventions. For example, dynamic monitoring can reduce the influence of patients' emotional changes, especially the white coat effect or masked hypertension on blood pressure measurement [20]. Wearable devices can track physical activity, monitor sleep patterns, and provide reminders for medication adherence. Mobile applications can offer diet and exercise tracking, stress management tools, and social support networks. Intelligent platforms can analyze patient data and provide personalized treatment plans based on individual needs and preferences [47–49].

Several studies have demonstrated the potential benefits of these technologies in improving health outcomes. For example, a recent small sample study conducted in the United States used mobile smart devices for monitoring and managing blood pressure at home in hypertensive patients and was found feasible and effective in reducing blood pressure [50]. While there is still much to learn about the effectiveness of these technologies in lifestyle medicine, the growing body of evidence suggests that they hold great promise for improving patient outcomes and reducing the burden of chronic disease.

### 4.6. Prospect

Currently, some lifestyle interventions for hypertension require larger and higher-quality research to determine their efficacy as antihypertensive treatments. For proven lifestyle modifications, long-term studies that span years or even decades are necessary to determine their long-term effects. The development of new technologies, such as artificial intelligence and mobile health platforms, shows promise in improving patient compliance, individualizing patient management, and ultimately enhancing the treatment outcomes for patients with hypertension.

## 5. Conclusions

Lifestyle medicine is an essential aspect of antihypertensive treatment and is considered as first-line therapy. Among lifestyle interventions, the most effective approach for reducing blood pressure may be diet modification, which includes low sodium intake and moderate alcohol consumption or abstinence. In addition, physical exercise and weight reduction are also recommended. Other lifestyle modifications, such as potassium supplementation, smoking cessation, and stress management, are not recommended by all five guidelines.

For patients with high-normal blood pressure or grade 1 hypertension without high risk factors, lifestyle medicine should be the initial treatment and should be continued for 3–6 months. If blood pressure is still uncontrolled, drug therapy can be initiated. For those patients who require medication, lifestyle modifications can enhance the effectiveness of antihypertensive treatment.

Longer-term studies are needed to determine the long-term effects of lifestyle modifications, and the use of new technologies, such as wearable devices and artificial intelligence, may enhance patient compliance and ultimately improve the treatment outcomes for hypertensive patients.

## Figures and Tables

**Table 1:** Summary list of recommendations for lifestyle medications in guidelines.

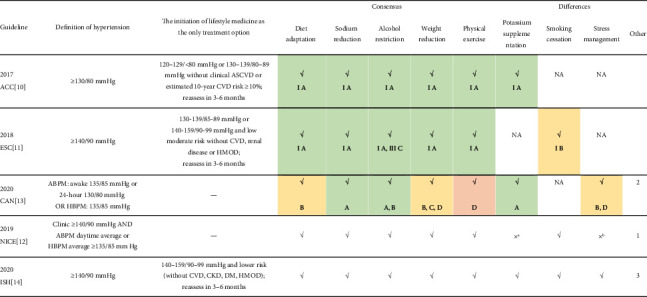

^a^Do not offer calcium, magnesium, or potassium supplements as a method for reducing blood pressure. ^b^NICE committee deleted the recommendation on relaxation therapies. 1: Discourage excessive consumption of coffee and other caffeine-rich products. 2: Calcium and magnesium intake. Supplementation of calcium and magnesium is not recommended for the prevention or treatment of hypertension (Grade B). 3: 3.1: Reduce exposure to air pollution and cold temperatures. 3.2: The use of complementary, alternative, or traditional medicines is not yet supported. 3.3: Moderate consumption of coffee, green tea, and black tea. Other beverages that can be beneficial include karkadé (hibiscus) tea, pomegranate juice, beetroot juice, and cocoa. NA: not available or recommended. Table green contains Grade A. Table yellow contains Grade B. Table red only contains Grade D.

**Table 2:** Summary of recommendations on diet adaptation in guidelines.

Guideline	Diet adaptation	Class of recommendation and level of evidence
2017 ACC [10]	A heart-healthy diet, such as the DASH (dietary approaches to stop hypertension) diet, that facilitates achieving a desirable weight is recommended for adults with elevated BP or hypertension. Consume a diet rich in fruits, vegetables, whole grains, and low-fat dairy products, with reduced content of saturated and total fat	I A
2018 ESC [11]	Increased consumption of vegetables, fresh fruits, fish, nuts, and unsaturated fatty acids (olive oil); low consumption of red meat; and consumption of low-fat dairy products are recommended	I A
2019 NICE [12]	Ask about people's diet and exercise patterns because a healthy diet and regular exercise can reduce blood pressure	NA
2020 CAN [13]	It is recommended that hypertensive patients and normotensive individuals at increased risk of developing hypertension consume a diet that emphasizes fruits, vegetables, low-fat dairy products, whole grain foods rich in dietary fibre, and protein from plant sources that is reduced in saturated fat and cholesterol (dietary approaches to stop hypertension) (DASH) diet	Grade B
2020 ISH [14]	Eating a diet that is rich in whole grains, fruits, vegetables, polyunsaturated fats, and dairy products and reducing food high in sugar, saturated fat, and trans fats, such as the DASH diet. Increase intake of vegetables high in nitrates known to reduce BP, such as leafy vegetables and beetroot	NA

**Table 3:** Summary of recommendations on sodium reduction in guidelines.

Guideline	Sodium reduction^*∗*^	Class of recommendation and level of evidence
2017 ACC [10]	Sodium reduction is recommended for adults with elevated BP or hypertension. Optimal goal is <1500 mg/d, but aim for at least a 1000 mg/d reduction in most adults	I A
2018 ESC [11]	Salt restriction to <5 g per day is recommended	I A
2019 NICE [12]	Encourage people to keep their dietary sodium intake low, either by reducing or substituting sodium salt, as this can reduce blood pressure	NA
2020 CAN [13]	To prevent hypertension and reduce BP in hypertensive adults, consider reducing sodium intake toward 2000 mg (5 g of salt or 87 mmol of sodium) per day	Grade A
2020 ISH [14]	Reduce salt added when preparing foods, and at the table. Avoid or limit consumption of high-salt foods such as soy sauce, fast foods, and processed food including breads and cereals high in salt	NA

^*∗*^1 mg sodium = 2.54 mg salt.

**Table 4:** Summary of recommendations of alcohol restriction in guidelines.

Guideline	Alcohol restriction	Class of recommendation and level of evidence
2017 ACC [10]	Adult men and women with elevated BP or hypertension who currently consume alcohol should be advised to drink no more than 2 and 1 standard drinks^*∗*^ per day, respectively	I A
	In individuals who drink alcohol, reduce alcohol to:	
	(i) Men: ≤2 drinks daily. (ii) Women: ≤1 drink daily	
	In the United States, 1 “standard” drink contains roughly 14 g of pure alcohol, which is typically found in 12 oz of regular beer (usually about 5% alcohol), 5 oz of wine (usually about 12% alcohol), and 1.5 oz of distilled spirits (usually about 40% alcohol)	

2018 ESC [11]	It is recommended to restrict alcohol consumption to:	I A
	(i) Less than 14 units per week for men. (ii) Less than 8 units per week for women	
	(1 unit is equal to 125 mL of wine or 250 mL of beer)	
	It is recommended to avoid binge drinking	III C

2019 NICE [12]	Ask about people's alcohol consumption and encourage a reduced intake if they drink excessively because this can reduce blood pressure and has broader health benefits	NA

2020 CAN [13]	In healthy adults, abstaining from alcohol or reducing alcohol intake to 2 drinks per day or less is recommended to prevent hypertension	Grade B
	In adults with hypertension who drink more than 2 drinks per day, a reduction in alcohol intake is associated with decreased BP and is recommended. In adults with hypertension who drink 6 or more drinks per day, a reduction in alcohol intake to 2 or fewer drinks per day is associated with decreased BP and is recommended	Grade A

2020 ISH [14]	Positive linear association exists between alcohol consumption, blood pressure, the prevalence of hypertension, and CVD risk. The recommended daily limit for alcohol consumption is 2 standard drinks for men and 1.5 for women (10 g alcohol/standard drink). Avoid binge drinking	NA

**Table 5:** Summary of recommendations about weight reduction in guidelines.

Guideline	Weight reduction	Class of recommendation and level of evidence
2017 ACC [10]	Weight loss is recommended to reduce BP in adults with elevated BP or hypertension who are overweight or obese	I A

2018 ESC [11]	Body weight control is indicated to avoid obesity (BMI >30 kg/m^2^ or waist circumference >102 cm in men and >88 cm in women), as is aiming at healthy BMI (about 20–25 kg/m^2^) and waist circumference values (<94 cm in men and <80 cm in women) to reduce BP and CV risk	I A

2019 NICE [12]	For guidance on the prevention of obesity and cardiovascular disease, see NICE's guidelines on obesity prevention and cardiovascular disease prevention	NA

2020 CAN [13]	(1) Height, weight, and waist circumference should be measured and body mass index calculated for all adults (Grade D)	Grade B, C, D
	(2) Maintenance of healthy body weight (body mass index 18.5–24.9 and waist circumference <102 cm for men and <88 cm for women) is recommended for nonhypertensive individuals to prevent hypertension (Grade C) and for hypertensive patients to reduce BP (Grade B). All overweight hypertensive individuals should be advised to lose weight (Grade B)	
	(3) Weight loss strategies should use a multidisciplinary approach that includes dietary education, increased physical activity, and behavioural intervention (Grade B)	

2020 ISH [14]	Body weight control is indicated to avoid obesity. Particularly abdominal obesity should be managed. Ethnic-specific cut-offs for BMI and waist circumference should be used. Alternatively, a waist-to-height ratio <0.5 is recommended for all populations	NA

**Table 6:** Summary of recommendations about physical exercise in guidelines.

Guideline	Physical exercise	Class of recommendation and level of evidence
2017 ACC [10]	Increased physical activity with a structured exercise program is recommended for adults with elevated BP or hypertension	I A
	(i) Aerobic: 90–150 min/wk, 65%–75% heart rate reserve	
	(ii) Dynamic resistance: 90–150 min/wk, 50%–80% 1 repetition maximum, 6 exercises, 3 sets/exercise, 10 repetitions/set	
	(iii) Isometric resistance: 4 × 2 min (hand grip), 1 min rest between exercises, 30%–40% maximum voluntary contraction, 3 sessions/wk, 8–10 wk	

2018 ESC [11]	Regular aerobic exercise (e.g., at least 30 min of moderate dynamic exercise on 5–7 days per week) is recommended	I A

2019 NICE [12]	Ask about people's diet and exercise patterns because a healthy diet and regular exercise can reduce blood pressure	NA

2020 CAN [13]	(i) For nonhypertensive individuals (to reduce the possibility of becoming hypertensive) or for hypertensive patients (to reduce their BP and to prescribe the accumulation of 30−60 minutes of moderate-intensity dynamic exercise) (e.g., walking, jogging, cycling, or swimming) on 4–7 days per week in addition to the routine activities of daily living	Grade D
	(ii) Higher intensities of exercise are not more effective	
	(iii) For nonhypertensive or hypertensive individuals with SBP/DBP of 140–159/90–99 mmHg, the use of resistance or weight training exercise (such as free-weight lifting, fixed-weight lifting, or handgrip exercise) does not adversely influence BP	

2020 ISH [14]	Studies suggest that regular aerobic and resistance exercise may be beneficial for both the prevention and treatment of hypertension. Moderate-intensity aerobic exercise (walking, jogging, cycling, yoga, or swimming) for 30 minutes on 5–7 days per week or HIIT (high-intensity interval training) which involves alternating short bursts of intense activity with subsequent recovery periods of lighter activity. Strength training also can help reduce blood pressure. Performance of resistance/strength exercises on 2-3 days per week	NA

**Table 7:** Summary of recommendations about potassium supplementation in guidelines.

Guideline	Potassium supplementation	Class of recommendation and level of evidence
2017 ACC [10]	Potassium supplementation, preferably in dietary modification, is recommended for adults with elevated BP or hypertension, unless contraindicated by the presence of CKD or the use of drugs that reduce potassium excretion	I A
	Aim for 3500–5000 mg/d, preferably by consumption of a diet rich in potassium	

2018 ESC [11]	NA	NA

2019 NICE [12]	Do not offer calcium, magnesium, or potassium supplements as a method for reducing blood pressure	NA

2020 CAN [13]	Potassium intake: in patients not at risk of hyperkalemia (see Table 4), increase dietary potassium intake to reduce BP	Grade A

2020 ISH [14]	Increase intake of other beneficial foods and nutrients including those high in magnesium, calcium, and potassium such as avocados, nuts, seeds, legumes, and tofu	NA

**Table 8:** Summary of recommendations about smoking cessation in guidelines.

Guideline	Smoking cessation	Class of recommendation and level of evidence
2017 ACC [10]	NA	NA
2018 ESC [11]	Smoking cessation, supportive care, and referral to smoking cessation programs are recommended	I B
2019 NICE [12]	Offer advice and help to smokers to stop smoking	NA
2020 CAN [13]	NA	NA
2020 ISH [14]	Smoking is a major risk factor for CVD, COPD, and cancer. Smoking cessation and referral to smoking cessation programs are advised	NA

**Table 9:** Summary of recommendations about stress management in guidelines.

Guideline	Stress management	Class of recommendation and level of evidence
2017 ACC [10]	NA	NA

2018 ESC [11]	NA	NA

2019 NICE [12]	Committee deleted the recommendation for relaxation therapies	NA

2020 CAN [13]	(1) In hypertensive patients in whom stress might be contributing to high BP, stress management should be considered as an intervention	Grade D
	(2) Individualized cognitive-behavioural interventions are more likely to be effective when relaxation techniques are used	Grade B

2020 ISH [14]	Chronic stress has been associated with high blood pressure later in life. Although more research is needed to determine the effects of chronic stress on blood pressure, randomized clinical trials examining the effects of transcendental meditation/mindfulness on blood pressure suggest that this practice lowers blood pressure. Stress should be reduced and mindfulness or meditation introduced into the daily routine	NA

## Data Availability

The data supporting the current study are available from the corresponding author upon request.
